# *Panax notoginseng* Alleviates Sepsis-Induced Acute Kidney Injury by Reducing Inflammation in Rats

**DOI:** 10.1155/2022/9742169

**Published:** 2022-05-21

**Authors:** Di-Wen Shou, Zi-Lin Yu, Jian-Biao Meng, Zhi-Zhen Lai, Li-Sha Pang, Mu-Hua Dai, Xue Yang, Yue-Xing Tu

**Affiliations:** ^1^The First Affiliated Hospital of Zhejiang Chinese Medical University (Zhejiang Provincial Hospital of Traditional Chinese Medicine), Hangzhou 310003, China; ^2^Emergency and Critical Care Center, Intensive Care Unit, Zhejiang Provincial People's Hospital (Affiliated People's Hospital, Hangzhou Medical College), Hangzhou 310014, China; ^3^Clinical Research Institute, Zhejiang Provincial People's Hospital (Affiliated People's Hospital, Hangzhou Medical College), Hangzhou 310014, China; ^4^Department of Critical Care Medicine, Tongde Hospital of Zhejiang Province, Hangzhou 310012, China

## Abstract

**Background:**

Sepsis is defined as a host inflammatory response to infection that can result in end-organ dysfunction. One of the most common consequences of sepsis is acute kidney injury (AKI). *Panax notoginseng* powder (PNP) has been previously reported to protect against overactive inflammation process. However, the potential effect of PNP on septic AKI is poorly described. The current study was conducted to investigate the protective effects of PNP in septic AKI rats.

**Methods:**

A model of septic AKI was established on male SD rats by using the cecal ligation and puncture procedure. PNP was administrated by gavage after the cecal ligation and puncture (CLP) procedure, and the mice were sacrificed at 6, 12, and 72 h after induction of sepsis. The serum and kidney samples were collected and assayed for biochemical tests, histopathological staining, inflammation, and apoptosis-related gene/protein expression. In addition, 15 rats in each group were used to calculate the 7-day survival rate.

**Results:**

CLP-induced kidney injury was observed by the histopathological score, which markedly was attenuated by PNP treatment. Consistently, PNP intervention significantly alleviated the elevated levels of serum creatinine and blood urea nitrogen in CLP-induced sepsis rats. The CLP procedure also triggered proinflammatory cytokine production and increased the expression of various inflammation-related proteins in the kidneys. However, PNP inhibited the renal expression of IL-18, IL-1*β*, TNF-*α*, and IL-6 to substantially improve inflammatory response. Mechanistically, CLP induced the increase of the NF-*κ*B p65 level in the injured kidneys, while PNP notably inhibited the corresponding protein expression.

**Conclusion:**

PNP attenuated kidney inflammation to protect against CLP-induced septic AKI in rats via inhibiting the NF-*κ*B signaling pathway.

## 1. Introduction

Sepsis can account for approximately 50% of acute kidney injury (AKI) cases [1, 2], and patients suffering from sepsis-associated AKI display a high risk of mortality which can lead to the development of chronic kidney disease (CKD) in the future [3]. Currently, volume resuscitation, antimicrobial therapy, and renal replacement therapy are the main therapeutic modalities used for the management of sepsis-induced AKI [4]. Despite advances in the treatment of sepsis, there is still no effective cure for septic AKI. Thus, exploring a new and novel approach for AKI treatment is essential.

With the development of traditional Chinese medicine (TCM) pathogenesis research, TCM treatment has proved to be immensely beneficial in the medical field [5–13]. Natural medications offer feasible alternative options against various diseases in contrast to western medication, primarily by effectively inhibiting antinociceptive as well as anti-inflammatory receptors [5, 6], and can also demonstrate both antitumor and antiviral activities [7, 8]. A number of previous studies have described the advantages of natural products in the treatment of various chronic diseases.

*Panax notoginseng* (Burk) F.H.Chen is derived from the dry root of the Araliaceae plant *Panax notoginseng*, which has been reported to exhibit diverse effects such as hemostasis, dissipating blood stasis, and reducing swelling as well as pain. A previous study has shown [14] that *Panax notoginseng* can exhibit a significant inhibitory effect on a variety of acute and chronic inflammation models of rats. It can effectively reduce capillary permeability and promote wound tissue repair. In addition, Xie et al. revealed [15] that *Panax notoginseng* can restore excessively high or low immune response to the normal level without interfering with the body's normal immune response. Moreover, *Panax notoginseng* can also substantially increase the function of the mononuclear and macrophage cells [16], promote the weight gain of immune organs, such as the thymus and spleen, and enhance the body's cellular and humoral immune functions. However, the potential effect of *Panax notoginseng* in AKI was still unclear.

In this study, we first used the cecal ligation and puncture procedure [17] to construct a sepsis-induced AKI model in rats and investigated the effect of *Panax notoginseng* powder (PNP) on pathological changes in the renal tissues and its role in regulating the renal function. Subsequently, the detailed molecular mechanisms including the expression of various inflammatory factors and signal pathways involved in PNP-treated AKI were also explored. Through these series of experiments, we aimed to reveal the protective effects of PNP on AKI in septic rats.

## 2. Materials and Methods

### 2.1. *Panax notoginseng* Powder

*Panax notoginseng*, also known as Sanqi, was prepared from the roots/radix of the herb *Panax notoginseng* that grows wild in East Asia, specifically China and Japan. *Panax notoginseng* powder was purchased from Zhejiang Provincial People's Hospital (Hangzhou, China) and stored in airtight containers for experiments.

### 2.2. Animal Experiments

Eighty male Sprague–Dawley rats (150–200 g) were obtained from the experimental animal center of Zhejiang Provincial People's Hospital and housed in a temperature- and light-controlled room (21–25°C and 12 h light-dark cycle) with arrangements for free drinking and eating. The rats were randomly divided into 4 different groups, 20 of each, including (1) the sham/control group with the rats receiving sham operation, (2) the sham/control + PNP group with the rats receiving sham operation and given gavage with PNP (8 mg/kg), (3) the CLP group with the rats being established as septic models by CLP, and (4) the CLP + PNP (8 mg/kg) group with the rats being established as septic models by CLP followed by gavage with PNP (8 mg/kg). The rats in sham + PNP and CLP + PNP (8 mg/kg) groups were given gavage with PNP according to the setting concentrations after surgery. After 6, 12, and 24 h of surgery, the blood was collected from the inner canthal orbital vein. The kidney tissue was thereafter divided into two and preserved in liquid nitrogen and formalin for fixation. The statistics of survival every day was analyzed until the seventh day. Ethical approval was approved by the local research ethics committee in Zhejiang Provincial People's Hospital, Affiliated People's Hospital of Hangzhou Medical College (Hangzhou, China).

### 2.3. Enzyme-Linked Immunosorbent Assay (ELISA)

The blood samples were collected in an anticoagulative tube with ethylenediaminetetraacetic acid (EDTA) and centrifuged at 3,000 *g* for 10 min. The supernatant and the serum were then separated and stored at 80°C for the detection of cytokines by ELISA within 3 days. According to the specifications of the ELISA kit (MultiSciences), the serum levels of the various cytokines of interest were detected with six replicates and three repeats in each. The absorbance values at 450 nm were measured and recorded. The standard curves of these cytokines were drawn, and the concentrations were calculated as per the manufacturer's instructions.

### 2.4. Assessment of BUN and Serum Creatinine (Scr)

The levels of BUN and Scr were examined with a biochemical analyzer (BX3010, Sysmex Corporation, Japan). The Scr level was measured by the picric acid method, and the BUN level was determined by the urease method.

### 2.5. Renal Histological Examination

The kidney tissues were fixed in 10% neutral buffered formalin, embedded in paraffin, and sectioned at 4 *μ*m thickness. After deparaffinization and rehydration, the kidney sections were stained with periodic acid-Schiff (PAS) or hematoxylin and eosin (HE). The sections were thereafter viewed by light microscopy at magnifications of x200 or x400. For semiquantitative analysis of the morphological changes, two different sections were randomly selected from each sample of at least 3 for every group and 10 fields were randomly selected at a magnification of x200 from each section. The various histopathological changes were evaluated by the percentage of injured/damaged renal tubules, as indicated by tubular lysis, dilation, disruption, and cast formation.

### 2.6. Immunohistochemical Staining

The kidney tissues were fixed in 10% neutral buffered formalin, embedded in paraffin, and sectioned at 4 *μ*m thickness. The sections were baked at 70°C for 2 h. Thereafter, the sections were deparaffinized in xylene, rehydrated using a gradient of ethanol concentrations, and boiled in 1 mM·TE buffer in a high-pressure cooker for 3 min to retrieve the antigen. They were blocked with 3% hydrogen peroxide for 15 min to inhibit the endogenous peroxidase activity and then incubated with 10% goat nonimmune serum (Invitrogen, Carlsbad, CA) for 20 min to reduce the nonspecific background staining. After that, TMA sections were incubated with rabbit antihuman primary polyclonal antibodies against IL-1*β*, TNF-*α*, and IL-6 (Cell Signaling Technology, CST) overnight at 4°C and then incubated with a biotin-labeled secondary antibody (Invitrogen, Carlsbad, CA) at room temperature for 15 min, followed by incubation with HRP-conjugated streptavidin (Invitrogen, Carlsbad, CA) at room temperature for 15 min. Then, the color development was performed with a DAB Substrate Kit (Dako, Glostrup, Denmark). Finally, the sections were counterstained with hematoxylin, dehydrated, cleared, and mounted.

### 2.7. Western Blot Analysis

Rat kidney cortexes were dissected and homogenized in radio immune precipitation (RIPA) lysis buffer (P0013B, Beyotime Biotechnology, Haimen, China). After centrifugation at 13,000 rpm for 15 min at 4°C, the supernatant was collected and the protein concentrations were analyzed using a bicinchoninic acid (BCA) Protein Assay Kit (Beyotime, Haimen, China). Bovine serum albumin (BSA) was used as the standard. Equal amounts of protein lysate were loaded directly on 10–12% SDS-PAGE and transferred onto the polyvinylidene difluoride (PVDF) membrane for protein blotting (0.2 *μ*m, Bio-Rad). The membranes were blocked with 5% BSA (w/v) in Tris-buffered saline with 0.1% Tween-20 (TBS-T) for 1 h at room temperature and thereafter incubated with indicated primary antibodies (Cell Signaling Technology, CST) overnight at 4°C. After being rinsed thrice with TBS-T at 5 min at 3 intervals, the membranes were further incubated with horseradish peroxidase-labeled goat anti-rabbit IgG (1 : 2000; Biosynthesis Biotechnology, Beijing, China) or goat anti-mouse IgG (1 : 2000; Biosynthesis Biotechnology, Beijing, China) for 1 h. The immunoblots were visualized using the Immobilon Western Chemiluminescent HRP Substrate (Millipore, Billerica, MA) with Bio-Rad ChemiDoc MP.

### 2.8. Statistical Analysis

All experiments were performed in triplicate unless otherwise stated. The data have been presented as mean ± SD. One-way analysis of variance (ANOVA) followed by the Student–Newman–Keuls (SNK) test was used for multiple group comparisons. The comparisons between the two groups were conducted using the two-tailed *t*-test, and *P* < 0.05 was considered statistically significant.

## 3. Results

### 3.1. PNP Treatment Improves Septic Rats' Survival Rate

**Figure 1:**
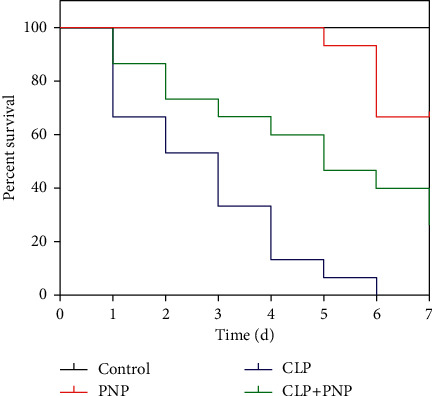
PNP treatment significantly improved survival in rats subjected to cecal ligation and puncture (CLP). Mortality was monitored twice daily for 7 days in control rats, septic (CLP alone) rats, and *Panax notoginseng* powder (PNP) (8 mg/kg)-treated septic rats. The rats were treated with PNP immediately after the CLP procedure. There were 15 rats in each group. The survival was evaluated using Kaplan–Meier analysis and the log-rank test.

First, we aimed to investigate the effect of PNP on the survival rate of septic rats with AKI. We compared the survival rate of rats treated with an early (immediately after CLP) dose of PNP. The rats were divided into four different groups, control, PNP, CLP, and CLP + PNP (*n* = 10, respectively), and were monitored twice a day for 7 days. As shown in Figure 1, rats that underwent the CLP procedure with no PNP treatment displayed a declining survival rate from the first day with a 50% survival rate on the next day. By the end of the experiment, no rats in the CLP group survived. In the PNP group, the survival rate of the rats reduced only 25% at the end of the experiment. In the CLP+PNP group, the survival rate of the rats was about 25% (shown in Figure 1); compared with the CLP group, there was a statistical difference (*P* < 0.01). These observations indicated that PNP treatment can significantly improve the survival rate of septic rats.

### 3.2. PNP Can Protect the Septic Rats against Renal Injury

**Figure 2:**
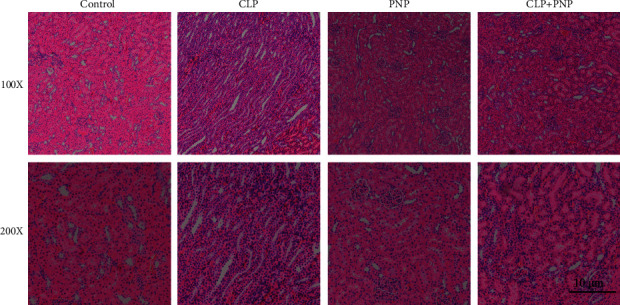
Effect of PNP on renal injury in septic rats. The CLP-induced histopathological changes in the kidney tissues were detected by an HE staining assay (magniﬁcation: 100x and 200x), and the renal injury score has been shown. No damage of tubules in control and PNP groups was observed. CLP induced severe tubular injury. The renal tubular damage caused by CLP was repaired by PNP administration. Scale bar: 10 *μ*m.

Next, we analyzed the histological changes caused by the CLP procedure and PNP treatment (8 mg/kg), and the results are shown in Figure 2. The pathological analysis of the CLP group showed increased inflammatory infiltration, evident edema, marked hemorrhagic spots in the interstitium, enlarged glomerular, narrower renal tubules, and degenerated epithelial cells (shown in Figure 2), thus suggesting that the CLP procedure induced sepsis in rats and caused substantial induced kidney injury in rats. However, compared to the control group and PNP group, the renal structure in the CLP + PNP group did not show significant changes associated with injury. These findings suggested that PNP treatment exhibited protective properties to sepsis-induced kidney injury in rats.

**Figure 3:**
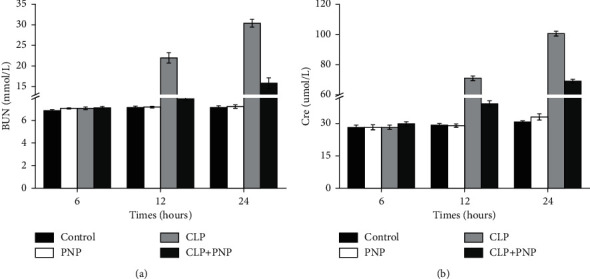
PNP alleviated CLP-induced kidney injury *in vivo*. The concentrations of BUN (a) and Cre (b) in the peripheral blood from the different groups are shown. Each value represents the mean ± SD.

We next analyzed the renal injury markers BUN and Cre in serum by ELISA, and the findings indicated that PNP treatment did not cause a statistically significant difference in the levels of both BUN and Cre, thereby indicating that PNP did not exhibit any obvious side effects on the renal tissues. Interestingly, the rats in the CLP group showed a high expression of BUN and Cre at 12 and 24 h (shown in Figures 3(a) and 3(b); *P* < 0.01), thus indicating that CLP induced impairment of renal function. Moreover, compared with the CLP model, the CLP + PNP group substantially reduced the increase of BUN (*P*=0.01) and Cre levels (*P* < 0.001).

### 3.3. PNP Alleviated Inflammation and Maintained the Normal Kidney Functions in Septic Rats

**Figure 4:**
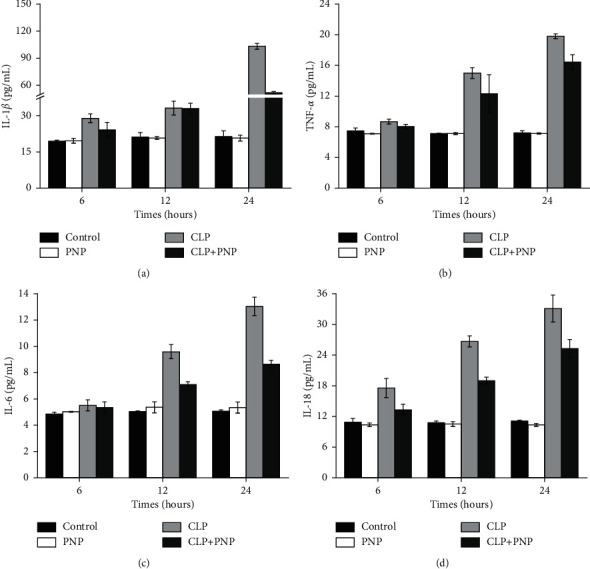
PNP markedly reduced the secretion of peripheral blood cytokines induced by CLP *in vivo*. IL-18, IL-1*β*, TNF-*α*, and IL-6 levels in kidney tissues were determined by ELISA. The results shown are representative of at least three independent experiments. Each value represents the mean ± SD.

After demonstrating the effects of PNP on the morphology of the renal tissues, we next explored whether PNP can also modulate the response of renal injury. The rats were divided into 4 different groups (control, PNP, CLP, and CLP + PNP), and the levels of various cytokines (IL-18, IL-1*β*, TNF-*α*, and IL-6) in the peripheral blood were measured. The data indicated that PNP had no obvious side effects on the renal tissues in rats. Compared to the control group, the levels of proinflammatory cytokines (IL-18, IL-1*β*, TNF-*α*, and IL-6) in rats in the CLP group were significantly increased (shown in Figures 4(a)–4(d); *P* < 0.01). Interestingly, compared with the CLP group, the CLP + PNP group showed reduced IL-18 (*P*=0.03), IL-1*β* (*P*=0.98), TNF-*α* (*P*=0.21), and IL-6 (*P*=0.02) levels at 12 h and caused a significant decrease in IL-18 (*P*=0.1), IL-1*β* (*P* < 0.001), TNF-*α* (*P*=0.02), and IL-6 (*P*=0.01) levels due to CLP at 24 h. These results confirmed the protective effects of PNP on inflammation at the molecular level.

**Figure 5:**
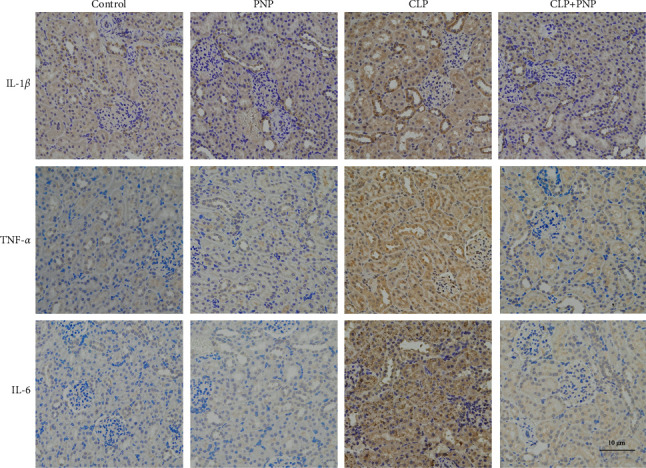
PNP attenuated the expression of cytokines in the kidney tissues induced by CLP *in vivo*. The expression of IL-1*β*, TNF-*α*, and IL-6 levels in kidney tissues was determined by immunohistochemical staining. The results shown are representative of at least three independent experiments. Scale bar: 10 *μ*m.

Next, immunohistochemistry was also used to evaluate the cytokine expression level of the various cytokines. A positive expression of IL-1*β* was observed in the CLP group, which was slightly higher than that of the control and PNP groups. This result indicated that an inflammatory response occurred in the CLP group, which then effectively induced an increased expression of the various cytokines. However, the inflammatory response caused by CLP decreased after PNP treatment, and the expression of cytokines was significantly downregulated (shown in Figure 5).

### 3.4. PNP Treatment Inhibited the NF-*κ*B Signaling Pathway in the Kidney Tissues of Septic Rats

**Figure 6:**
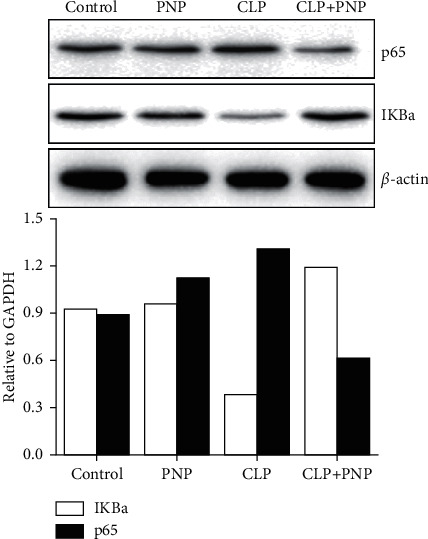
PNP treatment inhibited NF-*κ*B activation in the kidney tissues of septic rats. The rats were treated with PNP (8 mg/kg) or vehicle immediately after cecal ligation and puncture (CLP) or control operation (control). The representative blots of protein levels of nuclear and NF-*κ*B p65 and I*κ*B*α* in the kidneys were determined by western blot analysis.

PNP has been previously reported to reduce NF-*κ*B activation which can play a main role in inflammation. To understand the role of PNP in modulating renal tissue inflammation in the model of sepsis, we examined the effect of PNP on the NF-*κ*B/*p*65 expression. We compared the protein expression of key members of the NF-*κ*B/*p*65 signaling pathway after the CLP procedure with/without PNP treatment. The results of the western blot assay revealed that PNP alone did not significantly alter I*κ*B*α* and NF-*κ*B levels and translocation compared to control. The CLP group, as expected, showed a significantly decreased expression of I*κ*B*α* protein, thus suggesting the degradation of I*κ*B*α* and then subsequently the release of NF-*κ*B. However, PNP treatment could significantly reverse this process, thereby inhibiting I*κ*B*α* protein degradation and decreasing the NF-*κ*B level (shown in Figure 6). This finding revealed that PNP might modulate the inflammatory response of septic rats by suppressing the NF-*κ*B signaling pathway.

## 4. Discussion

In the present study, we have established a sepsis-induced AKI rat model via the CLP procedure to explore the potential protective effects of PNP. The CLP procedure induced pathological injury at the histological and molecular level in rat renal tissues. However, we observed that PNP treatment exerted a substantial protective effect on the kidneys of septic rats. We found that the infiltration of inflammatory cells of rates was reduced and the kidney injury was also attenuated after the PNP treatment. Moreover, we noticed that PNP treatment effectively reduced the levels of classical AKI markers BUN and creatinine indicating that PNP might act as a protective agent for maintaining normal renal functions in the sepsis-induced AKI rat model.

Sepsis is initiated by bacteria and toxins which can result in an overly activated systemic inflammatory response. Following this, immune response is activated, and without proper interventions, it may result in organ disorders [18]. Inflammatory cytokines, such as TNF-*α* and IL-6, have been demonstrated to be associated with poor outcomes in AKI [19]. Lipopolysaccharide is the main component of Gram-negative bacteria and can effectively bind to the pattern recognition receptor, TLR4. This binding can result in the activation of the nuclear factor-kappa B (NF-*κ*B) signaling pathway. The activation of this pathway can lead to increased transcription of pro-inflammatory cytokines such as TNF-*α*, IL‐1*β*, and IL-6 [20]. In addition, these cytokines can modulate the various transmembrane receptors, TLRs and inflammasome NLRs, and thereby amplify the initial inflammatory response [21, 22]. This whole process can thereafter result in excessive inflammation infiltration and may directly affect the renal parenchyma [20]. After ischemia/reperfusion, endothelial cells can increase the transcription level of a number of adhesion molecules which can in turn enhance the migration of leukocytes and might also amplify inflammatory response [23]. For instance, a study by Chen et al. demonstrated that inflammation and oxidative stress inhibition may be beneficial to kidney injury [24]. In our study, we observed that PNP significantly reduced the levels of proinflammatory factors IL-18, IL-1*β*, TNF-*α*, and IL-6. Tumor necrosis factor-alpha (TNF-*α*) can regulate the expression of various inflammation genes, oxidative stress, and antiapoptotic signaling pathways and thus act as one of the most important proinflammatory cytokines [25]. Thus, therapeutic targeting of TNF-*α* signaling has been extensively used for the treatment of several inflammatory diseases [26, 27]. TNF-*α* can induce the expression of IL-6, IL-10, and IL-18, which can be used as an inflammatory marker for the development of sepsis [28]. Consistent with this previous study, our present study also revealed that the protective role of PNP in AKI was mediated through inhibition of inflammatory responses.

In order to analyze the protective and anti-inflammatory effects of PNP in sepsis-induced AKI, we also investigated the effect on the key inflammatory transcription factor, NF-*κ*B. Activation of the NF-*κ*B/p65 signaling pathway has been reported as one of the key contributors of inflammation in kidney injury [29]. Usually, NF-*κ*B p65 remains in an inactive form in the cytoplasm when binding to I*κ*B*α*. I*κ*B*α* is phosphorylated by IKK and subsequently degraded via the ubiquitin-proteasome pathway after being stimulated by LPS, which results in the release of NF-*κ*B to induce the transcription of various inflammatory genes [24]. By using immunoblot analysis, we found a substantial decrease in p65 and an increase in I*κ*B*α* expression in septic mice kidneys upon PNP treatment compared with the control group. This finding indicated that PNP treatment can alleviate kidney injury through modulating the NF-*κ*B signaling pathway.

**Figure 7:**
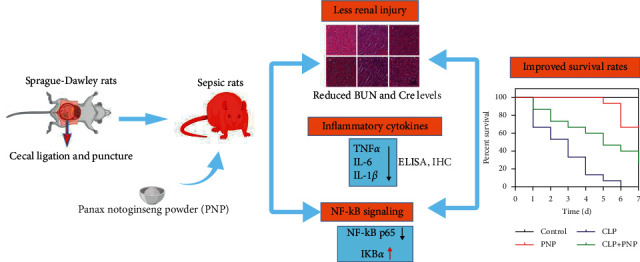
A schematic abstract depicting a summary of the study design and results. Sprague–Dawley rats undergoing the CLP procedure developed sepsis and AKI. PNP significantly reduced renal injury to a lesser degree accompanied with reduced BUN/Cre levels, thereby indicating improved kidney function. PNP primarily exerted its protective effects against kidney injury by inhibiting the inflammatory cytokine production and NF-*κ*B expression. Ultimately, PNP improved the survival rate of septic rats, thus displaying its potential as a therapeutic agent in sepsis-induced AKI.

The limitation of this paper is that we found that *Panax notoginseng* powder caused a certain reduction in the survival rate of rats in the sham-operated group. According to the previously published literature [30, 31], we found that *Panax notoginseng* has certain toxic and side effects, mainly on the heart, which may be the reason for the death of rats as shown in Figure 1. Therapeutic drugs have toxic and side effects at the same time of treatment, which is a problem that should be comprehensively considered. In conclusion, as depicted in the schematic diagram (shown in Figure 7), we demonstrated that PNP protected the kidney functions and alleviated inflammation through modulating the NF-*κ*B p65 signaling pathway in the rats with sepsis-induced acute kidney injury. Therefore, PNP might be considered as one of the potential therapeutic drugs to target sepsis-induced AKI. However, the detailed mechanisms through which PNP can modulate the NF-*κ*B signaling pathway and immune functions to mitigate AKI will also need to be investigated in the near future, and we will further optimize the dose and concentration of PNP in order to avoid side effects as far as possible in the near future and pay attention to the indicators of the heart.

## Data Availability

The data that support the findings of this study can be made available from the corresponding author Yue-Xing Tu upon reasonable request.
